# Safety and effectiveness of selpercatinib in patients with *RET* fusion-positive non-small cell lung cancer in real-world clinical practice: a postmarketing study in Japan

**DOI:** 10.1093/jjco/hyaf220

**Published:** 2026-01-25

**Authors:** Yucherng Chen, Joji Mori, Satoshi Wakabayashi, Kevin Y Urayama

**Affiliations:** Safety Medical, Global Patient Safety & Solutions, Eli Lilly Japan K.K., 5-1-28, Isogamidori, Chuo-ku, Kobe, Hyogo, 651-0086, Japan; Global Statistical Science Japan, Eli Lilly Japan K.K., 5-1-28, Isogamidori, Chuo-ku, Kobe, Hyogo, 651-0086, Japan; Japan Drug Development & Medical Affairs, Eli Lilly Japan K.K., 5-1-28, Isogamidori, Chuo-ku, Kobe, Hyogo, 651-0086, Japan; Pharmacoepidemiology, Global Patient Safety & Solutions, Eli Lilly Japan K.K., 5-1-28, Isogamidori, Chuo-ku, Kobe, Hyogo, 651-0086, Japan

**Keywords:** effectiveness, NSCLC, postmarketing, *RET* fusion-positive, safety, Selpercatinib

## Abstract

**Background:**

This study assessed the safety and effectiveness of selpercatinib, a selective rearranged during transfection (*RET)* kinase inhibitor, in patients with *RET* fusion-positive non-small cell lung cancer (NSCLC) in real-world clinical practice in Japan.

**Methods:**

This single-arm, multicenter, prospective observational study included patients with *RET* fusion-positive NSCLC who received at least one dose of selpercatinib between February 2022 and October 2023. Data were extracted from medical records and entered into an electronic data capture (eDC) system. Safety (adverse events [AEs] and AEs of special interest [AESIs]) and effectiveness (tumor response, overall survival [OS]) were assessed over 12 months.

**Results:**

Among 243 patients (median age: 67 years; 56% females), AEs occurred in 86.0% of patients, with Grade ≥ 3 AEs in 48.1% and serious AEs (SAEs) in 24.7%. The most common AEs (≥10%) included hypertension (23.5%), abnormal hepatic function (21.0%), rash (11.1%), aspartate aminotransferase increase (10.3%), and diarrhea (10.3%). AEs led to treatment modifications, including dose interruption (54.7%), dose reduction (14.8%), and discontinuation (6.2%). AESIs included liver injury (44.0%), hypertension-related events (23.9%), and hypersensitivity (MedDRA preferred term; 9.9%). The overall response rate was 58.8%, comprising complete response in 4.5% and partial response in 54.3% of patients. Median OS was not reached; the 12-month survival rate was 80.9% (95% CI, 74.9–85.6).

**Conclusions:**

Real-world data showed selpercatinib to be effective in patients with *RET* fusion-positive NSCLC in Japan, with a favorable safety profile and no new safety concerns.

## Graphical Abstract

**Graphical Abstract:**
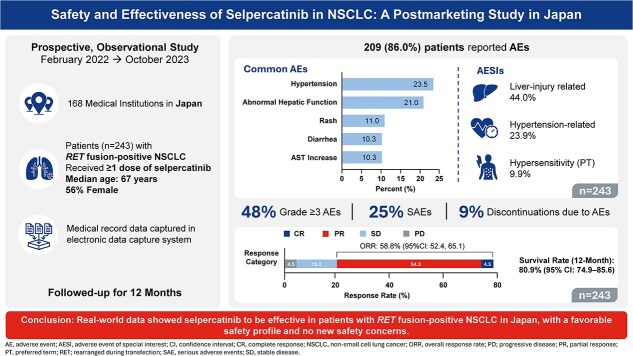

## Introduction

Lung cancer is the most prevalent and lethal malignancy worldwide, responsible for an estimated 2.5 million new cases and 1.8 million deaths annually [1]. In Japan, it is the second most prevalent malignancy after colorectal cancer, accounting for 13.6% of all cancer cases and 83 243 deaths in 2022 [2]. Non-small cell lung cancer (NSCLC), which represents ~85% of all lung cancer cases, is often diagnosed at an advanced stage when the disease is typically unresectable. This delayed diagnosis leads to poor prognosis for stage IV patients, with a 5-year survival rate only as high as 10% [3].

NSCLC manifests through various oncogenic drivers, including alterations in the rearranged during transfection (*RET*) receptor, a transmembrane receptor tyrosine kinase important for normal tissue development and maintenance [4]. Though rare, appearing only in 1%–2% of patients with NSCLC (primarily adenocarcinoma), *RET* fusions have emerged as critical drivers of tumor progression [5, 6]. The discovery of *RET* fusions in NSCLC has significantly transformed treatment strategies enabling the development of targeted therapies and underscoring the importance of comprehensive testing and personalized treatment for patients with these genomic alterations [7–10].

Selpercatinib, a first-in-class, highly selective *RET* kinase inhibitor [11], is approved in Japan [12] and other countries [13] for *RET* fusion-positive NSCLC, advanced or relapsed solid tumors, and *RET* mutant unresectable medullary thyroid cancer. Phase 1/2 and phase 3 clinical trials have demonstrated its efficacy in treating *RET* fusion-positive NSCLC and has shown a manageable safety profile [14, 15]. A global phase 1/2 study reported the most common Grade ≥ 3 treatment-emergent adverse events (TEAEs) to be hypertension (19.7%), increased alanine aminotransferase (ALT; 11.4%), increased aspartate aminotransferase (AST; 8.8%), diarrhea (5.0%), and QT prolongation (4.8%) [14]. A phase 3 study reported similar findings, with increased ALT (22.0%), hypertension (20.0%), increased AST (13.0%), and QT prolongation (9%) as the most common Grade ≥ 3 TEAEs [15].

In a Japanese subgroup from the phase 1/2 trial, selpercatinib showed an overall response rate (ORR) of 55.3%, with liver injury, QT prolongation, hypersensitivity, and hypertension identified as key safety risks [16]. In Japan, epiphysiolysis in adolescents and interstitial lung disease (ILD) were also identified as important risks [17]. Other important potential risks include bleeding, abnormal bone growth, fetal embryo toxicity, and use of selpercatinib in patients with impaired liver function as outlined in the Japanese Risk Management Plan [17–19]. Among these, hypersensitivity reactions, occurring in ~6%–7% of patients, have emerged as a potential clinical interest, particularly in those previously treated with immune checkpoint inhibitors (ICIs) [13, 19, 20]. Selpercatinib-associated hypersensitivity reactions typically manifest within the initial weeks of therapy and are often characterized by maculopapular rash, commonly preceded by fever and accompanied by arthralgias or myalgias, differing from acute hypersensitivity reactions such as anaphylaxis or angioedema [20–22]. Considering the relevance of hypersensitivity to selpercatinib’s safety profile, its patterns in clinical studies warrant further investigation, particularly in real-world settings.

Given the limited safety and efficacy data of selpercatinib in Japanese patients and the rarity of *RET* fusion-positive NSCLC, the real-world impact of selpercatinib in Japan remains unclear. Therefore, a postmarketing safety study (PMSS) nearly capturing all patients with *RET* fusion-positive NSCLC in Japan treated with selpercatinib in real-world routine clinical practice was conducted to evaluate the safety and effectiveness of this drug. This study was carried out in compliance with requirements from the Japanese Pharmaceuticals and Medical Devices Agency.

## Patients and methods

### Patients and study design

This single-arm, multicenter, prospective observational study was conducted in patients diagnosed with *RET* fusion-positive NSCLC who received at least one dose of selpercatinib between February 2022 and October 2023 in the setting of routine clinical practice across 168 medical institutions in Japan. Patients participating in any other clinical trials were excluded.

This was an all-case PMSS supported by a regulatory mandate to identify and attempt registration of all eligible patients across Japan. The physicians pursued recruitment of eligible patients and provided registration information through a centralized electronic data capture (eDC) system or through a paper registration form.

Patients were observed for up to 12 months from the start of selpercatinib treatment (index date). For safety outcomes, all patients were followed until the earliest occurrence of 30 days after the last selpercatinib dose, initiation of subsequent anti-tumor treatment, completion of the 12-month follow-up period, loss to follow-up, or death. For the survival outcome, patients were followed for up to 12 months, regardless of discontinuation.

The study was conducted in accordance with the Good Post-Marketing Study Practice Ordinance in Japan (GPSP Ordinance, Ministry of Health, Labor and Welfare Ordinance No. 171, dated 20 December 2004). In accordance with these regulations, neither independent review board and ethics committee approval nor patient consent were required. All data used in this study were anonymized.

### Data collection and assessments

Data were obtained from patient’s medical records under routine clinical practice and entered through the eDC system based on an electronic case report form (eCRF). The eDC system was designed to securely collect and transmit patient data from participating medical institutions via the internet. When the eDC system was unavailable, paper CRFs were used.

Baseline patient characteristics included age, sex, tumor type, Eastern Cooperative Oncology Group performance status (ECOG PS), smoking status, medical history, comorbidities, chest computed tomography (CT), electrocardiogram (ECG) findings, and Child-Pugh classification (for impaired liver function). Additionally clinical data pertaining to the cancer diagnosis collected at baseline included histological subtype, cancer stage, recurrence status, sites of metastasis or recurrence, tumor invasion of major chest arteries, airway exposure, and programmed death-ligand 1 (PD-L1) expression status. A history of radiation therapy, surgery, systemic anti-cancer treatments, and ICI use was recorded prior to the initiation of selpercatinib treatment.

Selpercatinib administration details, including start and end dates, dosage frequency, dose reductions, drug interruptions, and treatment discontinuations, were collected based on prescription records provided by the treating physicians. Concomitant therapy data, including cancer treatments, drugs for AEs or other medical conditions, radiation therapy, and surgeries, were also collected at baseline and during selpercatinib administration (if available).

Laboratory assessments included a chemistry profile, liver function tests that measure ALT, AST, alkaline phosphatase (ALP), gamma-glutamyl transferase (γ-GTP), total bilirubin, and direct bilirubin. Additionally, albumin, total protein levels, and coagulation parameters (prothrombin time-international normalized ratio [PT-INR] and activated partial thromboplastin time [APTT]) were assessed. These tests were performed at baseline and at the time of AEs.

#### Safety

Safety data, including the incidence, grade, and severity of AEs were collected throughout the observation period. AEs of special interest (AESIs) included liver injury, QT interval prolongation, hypertension, hemorrhage, interstitial lung disease, and hypersensitivity (defined as Medical Dictionary for Regulatory Activities (MedDRA) preferred term [PT] “Hypersensitivity” and “drug hypersensitivity”). Regarding hypersensitivity, additional events not formally reported as AEs, but identified as hypersensitivity-related based on the physician’s judgment, were captured in the eCRF and considered as hypersensitivity-related events. AEs, AESIs, and serious AEs (SAEs) were classified using MedDRA (Version 27.0) and summarized by system-organ class and preferred terms. The grading of the safety events was based on the National Cancer Institute Common Terminology Criteria for Adverse Events (CTCAE, Version 5.0).

#### Effectiveness

Effectiveness was assessed based on tumor response and overall survival (OS). The best tumor response during the first 6 months and 12 months following the initial selpercatinib dose was evaluated using the physician-assessed tumor response according to Response Evaluation Criteria in Solid Tumors (RECIST) 1.1 criteria [23]. Tumor responses were categorized as complete response (CR), partial response (PR), stable disease (SD), or progressive disease (PD), with the ORR also reported. OS was defined as the time from the first selpercatinib administration to death from any cause. Following selpercatinib discontinuation, survival status was tracked for up to 12 months from treatment initiation.

### Statistical analysis

Except for OS, all other data points were presented using descriptive statistical analyses. Safety data were summarized by body system and preferred terms, with AEs, AESIs, and SAEs presented as frequency tables for categorical variables (frequency and incidence proportions) and summary statistics (mean, median, standard deviation) for continuous variables.

The ORR was estimated based on the proportion of patients who had the best overall response of CR and PR, and this percentage is shown together with 95% confidence interval (CI). OS was descriptively summarized using the Kaplan–Meier method. Median (95% CI) of OS and survival rate (%) at specified time points (3, 6, 9, and 12 months) were estimated.

## Results

### Patient disposition, demographics, and baseline characteristics

**Figure 1:**
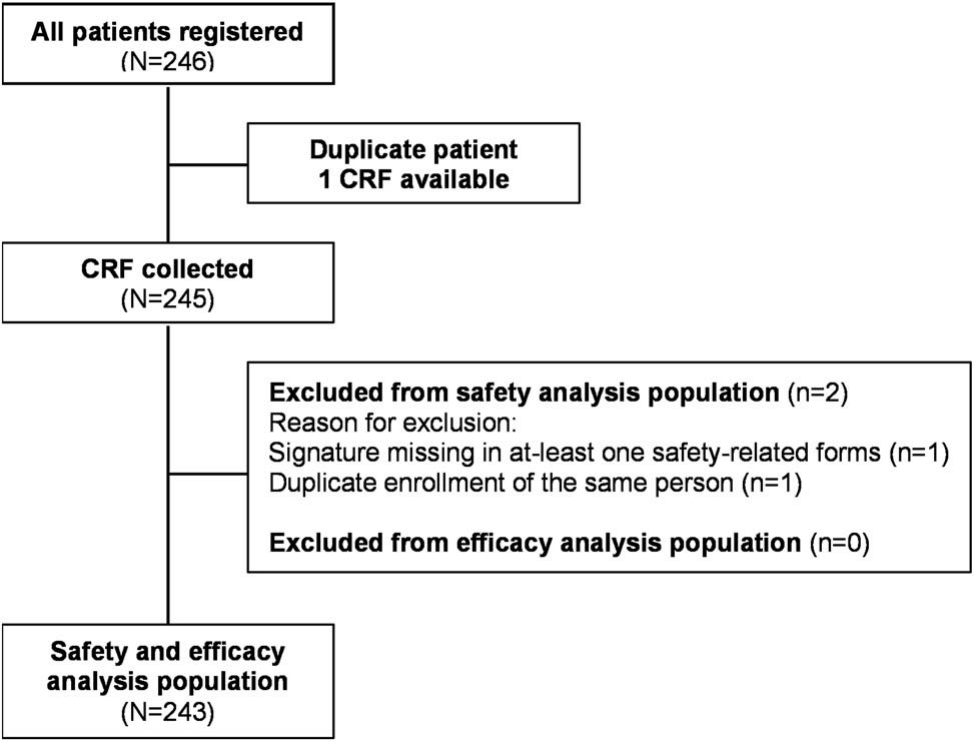
Patient disposition CRF, case report form.

A total of 246 patients were initially enrolled, of which 243 constituted the final safety and effectiveness analysis population **(**Fig. 1**)**. The median (min-max) age of the patients was 67 (29–93) years, with 24.7% (n = 60) aged ≥75 years; and 56% (n = 136) were female.

More than 62% (n = 151) of patients had never smoked. Most patients had ECOG PS of 0 (45.3% [n = 110]) or 1 (43.6% [n = 106]). The majority (84.0% [n = 204]) of patients had stage IV NSCLC, with lymph nodes being the most common metastatic site (49.0% [n = 119]) **(**Table 1**)**. Overall, 38.3% (n = 93) of patients had not received any prior treatment, while 61.7% (n = 150) had received some form of anti-cancer therapy before enrollment.

**Table 1:** Patient demographics and baseline characteristics.

**Characteristics**	**Analysis population (N = 243)**
**Sex, n (%)**	
Male	107 (44.0)
Female	136 (56.0)
**Age, years**	
Mean (SD)	65.8 (12.2)
Median (range)	67.0 (29–93)
**Age groups (years), n (%)**	
<65	103 (42.4)
≥65 to <75	80 (32.9)
≥75	60 (24.7)
**Smoking history, n (%)**	
Current	4 (1.6)
Former	86 (35.4)
Never	151 (62.1)
Unknown	2 (0.8)
**ECOG PS at baseline, n (%)**	
0	110 (45.3)
1	106 (43.6)
2	16 (6.6)
**Current comorbidities** ^**a**^ **, n (%)**	182 (74.9)
Hypertension	99 (40.7)
Liver injuries (Child-Pugh score class A)	10 (4.1)
QTc interval prolongation	3 (1.2)
Hemorrhage	3 (1.2)
Hypersensitivity	2 (0.8)
Renal injury	7 (2.9)
**Primary or recurrent tumor, n (%)**	
Primary	145 (59.7)
Recurrence	98 (40.3)
**Histological/Pathological type, n (%)**	
Squamous cell carcinoma	2 (0.8)
Non-squamous cell carcinoma	239 (98.4)
Others	2 (0.8)
**Stage, n (%)**	
Stage IA-IIB	8 (3.3)
Stage IIIA	13 (5.3)
Stage IIIB	11 (4.5)
Stage IIIC	2 (0.8)
Stage IV	204 (84.0)
Unknown	5 (2.1)
**Site of metastases** ^b^ **,** ^**n (%)**^	
Lymph nodes	119 (49.0)
Lung	98 (40.3)
Bone	90 (37.0)
Pleural (with pleural effusion)	84 (34.6)
Brain	55 (22.6)
Liver	39 (16.0)
Pleural (without pleural effusion)	29 (11.9)
Local	29 (11.9)
Others	35 (14.4)
**PD-L1 status, n (%)**	
Strong positive	83 (34.2)
Weak positive	79 (32.5)
Negative	50 (20.6)
Unknown	31 (12.8)
**ECG conducted, n (%)**	211 (86.8)
**ECG abnormal findings** ^**c**^ ^**,**^ ^**d**^ **, n (%)**	
Yes	33 (15.6)
No	178 (84.4)
**QTcF interval groups** ^**c**^ ^**,**^ ^**d**^ **, n (%)**	
<450 msec	203 (96.2)
<470 msec	209 (99.1)
≥470 msec	2 (0.9)
**QTcF interval** ^**c**^ ^**,**^ ^**d**^ **,(msec)**	
Mean (SD)	410.9 (24.5)
Median (range)	412.0 (325–484)
**Chest CT performed, n (%)**	
Yes	235 (96.7)
**Interstitial shadow** ^**e**^ ^**,**^ ^**f**^ **, n (%)**	
Yes	10 (4.3)
**Prior therapies for NSCLC, n (%)**	
Surgical procedure	69 (28.4)
Radiotherapy	77 (31.7)
History of anti-cancer therapy	150 (61.7)
Number of regimens prior to selpercatinib, n (%)	
None	93 (38.3)
1	55 (22.6)
2	39 (16.0)
≥3	56 (23.0)
ICI therapy, n (%)	93 (38.3)
Anti-PD-1 or anti-PD-L1 therapy only	90 (37.0)
Both of anti-PD-1 or anti-PD-L1 therapy and anti- CTLA-4 therapy	3 (1.2)
Period from last administration of ICI to selpercatinib initiation^g^^,^^h^, n (%)	
<3 months	15 (16.1)
≥3 months and < 6 months	9 (9.7)
≥6 months	69 (74.2)

Abbreviations: CT, computed tomography; CTLA-4; Cytotoxic T-lymphocyte-associated protein 4; ECG, electrocardiogram; ECOG PS, Eastern Cooperative Oncology Group performance status; ICI, immune checkpoint inhibitor; msec, millisecond; N, number of patients; n, number of patients in the specified category; NSCLC, non-small cell lung cancer; PD-1, Programmed death-1; PD-L1, programmed death-ligand 1; QTc, corrected QT interval; QTcF, QT interval corrected by Fridericia's method; *RET*, rearranged in transfection; SD, standard deviation.

^a^Patients may have multiple comorbidities

^b^Multiple sites are allowed. Not mutually exclusive.

^c^The number of “ECG performed is Yes” is used as the denominator.

^d^N = 211.

^e^The number of “Chest CT performed is Yes” is used as the denominator.

^f^N = 235.

^g^Used the number of “History of receiving any ICI is Yes” as a denominator.

^h^N = 93.

### Selpercatinib treatment

Selpercatinib was generally administered at the recommended dose (160 mg twice daily), with a median relative dose intensity (RDI) of 73.8%. Most patients (72.0% [n = 175]) received treatment for 6 to 12 months. More than half of the patients had selpercatinib doses reduced from the initial dose (55.6% [n = 135]), including 43.7% (n = 59) of patients with ≥2 dose reductions. Of these, 60.0% (n = 81) of patients had their dose re-escalated by at least one level following a dose reduction **(**Supplementary Table S1**)**. Over 12 months, 36.2% (n = 88) of patients discontinued selpercatinib, primarily due to PD (56.8% [n = 50]), loss to follow-up (20.5% [n = 18]), and AEs (17.0% [n = 15]).

### Safety

Of the 243 patients, 86.0% (n = 209) experienced at least one AE **(**Table 2**)**. The most frequently reported AEs (MedDRA PT, any grade, occurring in ≥10% of patients) were hypertension (23.5% [n = 57]), abnormal hepatic function (21.0% [n = 51]), rash (11.1% [n = 27]), and AST increase and diarrhea (25 each [10.3%] patients) **(**Table 2**)**. Approximately 60% of AEs had recovered or were recovering during the observation period of this study.

**Table 2:** Summary of adverse events, serious adverse events, and Grade ≥ 3 adverse events^a^.

**Preferred Term** ^b^	**AEs, n (%)**	**Grade ≥ 3, n (%)**	**SAEs, n (%)**
Safety analysis set	N = 243		
Patient with ≥1 event	209 (86.0)	117 (48.1)	60 (24.7)
Hypertension	57 (23.5)	23 (9.5)	2 (0.8)
Hepatic function abnormal	51 (21.0)	37 (15.2)	18 (7.4)
Rash	27 (11.1)	3 (1.2)	2 (0.8)
AST increased	25 (10.3)	7 (2.9)	4 (1.6)
Diarrhea	25 (10.3)	1 (0.4)	2 (0.8)
Hypersensitivity	24 (9.9)	8 (3.3)	9 (3.7)
ALT increased	24 (9.9)	12 (4.9)	3 (1.2)
Pyrexia	22 (9.1)	1 (0.4)	0.0
Platelet count decreased	20 (8.2)	5 (2.1)	2 (0.8)
Edema peripheral	16 (6.6)	1 (0.4)	0.0
Electrocardiogram QT prolonged	16 (6.6)	4 (1.6)	1 (0.4)
Dry mouth	15 (6.2)	1 (0.4)	1 (0.4)
Blood creatinine increased	15 (6.2)	1 (0.4)	1 (0.4)
Liver disorder	14 (5.8)	4 (1.6)	0.0

Abbreviations: AE, adverse event; ALT, alanine aminotransferase; AST, aspartate aminotransferase; MedDRA, Medical Dictionary for Regulatory Activities; SAE, serious adverse event.

^a^Shown are AEs that occurred in at least 5% of the patients.

^b^The terms used to describe the AEs are adapted from or composites of MedDRA Version 27.0, preferred terms.

Grade ≥ 3 AEs occurred in 48.1% (n = 117) of patients. The most common (occurring in ≥3% of patients) Grade ≥ 3 AEs were abnormal hepatic function (15.2% [n = 37]), hypertension (9.5% [n = 23]), ALT increase (4.9% [n = 12]), and hypersensitivity (MedDRA PT; 3.3% [n = 8]) **(**Table 2**)**. Overall, 24.7% (n = 60/243) of patients experienced SAEs. The most frequently reported SAEs (occurring in ≥3% of patients) were abnormal hepatic function (7.4% [n = 18]) and hypersensitivity (3.7% [n = 9]) **(**Table 2**)**.

A total of 6.2% (n = 15) of patients discontinued treatment due to AEs, including 2.5% (n = 6) due to SAEs **(**Table 3**)**. In total, 54.7% (n = 133) of patients had treatment interruption, and 36 (14.8%) had dose reductions due to AEs.

**Table 3:** Adverse events leading to dose interruption^a^ and treatment discontinuation.

	**Dose interruption**		**Treatment discontinuation**	
**Preferred term** ^b^	**AEs, n (%)**	**SAEs, n (%)**	**AEs, n (%)**	**SAEs, n (%)**
Safety analysis set	N = 243			
Patients with ≥1 event	133 (54.7)	42 (17.3)	15 (6.2)	6 (2.5)
Hepatic function abnormal	32 (13.2)	12 (4.9)	3 (1.2)	3 (1.2)
Hypersensitivity	23 (9.5)	9 (3.7)	1 (0.4)	0
Pyrexia	12 (4.9)	0	–	–
Hypertension	11 (4.5)	1 (0.4)	–	–
Rash	10 (4.1)	2 (0.8)	–	–
AST increased	9 (3.7)	3 (1.2)	1 (0.4)	1 (0.4)
Electrocardiogram QT prolonged	8 (3.3)	1 (0.4)	–	–
Liver disorder	6 (2.5)	0	–	–
ALT increased	5 (2.1)	2 (0.8)	1 (0.4)	–
Decreased appetite	4 (1.6)	1 (0.4)	2 (0.8)	1 (0.4)
Stomatitis	4 (1.6)	0	2 (0.8)	0
Edema peripheral	4 (1.6)	0	–	–
Neutrophil count decreased	4 (1.6)	0	–	–
Thrombocytopenia	3 (1.2)	3 (1.2)	–	–
Rash maculopapular	3 (1.2)	0	–	–
Malaise	3 (1.2)	0	–	–
Blood creatinine increased	3 (1.2)	1 (0.4)	–	–
Platelet count decreased	3 (1.2)	1 (0.4)	–	–
Generalized edema	1 (0.4)	0	1 (0.4)	–
Lung disorder	–	–	1 (0.4)	1 (0.4)
Syncope	–	–	1 (0.4)	–
Corneal disorder	–	–	1 (0.4)	–
Fatigue	–	–	1 (0.4)	–

Abbreviations: AE, adverse event; ALT, alanine aminotransferase; AST, aspartate aminotransferase; MedDRA, Medical Dictionary for Regulatory Activities; QT, SAE, serious adverse event.

^a^Shown are AEs that occurred in at least 1% of the patients.

^b^The terms used to describe the AEs are adapted from or composites of MedDRA Version 27.0, preferred terms.

A total of 3 deaths due to sepsis, bacterial pleurisy, and hemoptysis were reported. Sepsis and bacterial pleurisy were assessed as not related to selpercatinib, while hemoptysis was considered related, but confounded by tumor exposure to the major airway.

#### Adverse events of special interest

##### Liver injury

Overall, 44.0% of patients (n = 107/243) experienced at least one liver injury-related AE. Apart from abnormal hepatic function (21.0% [n = 51]), notable liver injury-related events (occurring in ≥5% of patients) included increased AST (10.3% [n = 25]), increased ALT (9.9% [n = 24]), and liver disorder (5.8% [n = 14]). In all, 10.3% (n = 25) of patients experienced liver injury-related SAEs, and 24.7% (n = 60) of patients experienced Grade ≥ 3 AEs **(**Table 4**)**. Among those with Grade ≥ 3 liver injury-related AEs, the majority (71.7% [n = 43]) required dose interruptions, while only 5.0% (n = 3) of patients had dose reductions without interruption **(**Supplementary Table S2**)**. Four (3.7%) patients discontinued treatment due to liver injury-related AEs. Notably, nearly 90% (n = 96) of these AEs had recovered during the observation period.

**Table 4:** Adverse events of special interest^a^.

**Preferred term** ^b^	**AEs, n (%)**	**Grade ≥ 3, n (%)**	**SAEs, n (%)**
Safety analysis set	N = 243		
**Liver injury-related AEs**			
Patients with ≥1 event	107 (44.0)	60 (24.7)	25 (10.3)
Hepatic function abnormal	51 (21.0)	37 (15.2)	18 (7.4)
AST increased	25 (10.3)	12 (4.9)	4 (1.6)
ALT increased	24 (9.9)	7 (2.9)	3 (1.2)
Liver disorder	14 (5.8)	4 (1.6)	0.0
Blood bilirubin increased	4 (1.6)	0.0	0.0
Hepatic enzyme increased	4 (1.6)	2 (0.8)	1 (0.4)
GGT increased	3 (1.2)	1 (0.4)	0.0
Blood ALP increased	3 (1.2)	1 (0.4)	0.0
**Hypersensitivity (PT)**			
Patients with ≥1 event	24 (9.9)	8 (3.3)	9 (3.7)
**Hypersensitivity-related events** ^**c**^			
Patients with ≥1 event	60 (24.7)	22 (9.1)	17 (7.0)
Rash	14 (5.8)	3 (1.2)	2 (0.8)
Hepatic function abnormal	13 (5.3)	9 (3.7)	5 (2.1)
Pyrexia	10 (4.1)	0.0	0.0
Platelet count decreased	6 (2.5)	1 (0.4)	0.0
ALT increased	3 (1.2)	0.0	0.0
Rash maculopapular	3 (1.2)	0.0	0.0
**QT Interval Prolongation**			
Patients with ≥1 event	16 (6.6)	4 (1.6)	1 (0.4)
Electrocardiogram QT prolonged	16 (6.6)	4 (1.6)	1 (0.4)
**Hypertension-related AEs**			
Patients with ≥1 event	58 (23.9)	24 (9.9)	2.0 (0.8)
Hypertension	57 (23.5)	23 (9.5)	2.0 (0.8)
Blood pressure increased	1 (0.4)	1 (0.4)	0.0
**Hemorrhage**			
Patients with ≥1 event	5 (2.1)	2 (0.8)	2 (0.8)
Epistaxis	1 (0.4)	0.0	0.0
Hemoptysis	1 (0.4)	1 (0.4)	1 (0.4)
Melaena	1 (0.4)	1 (0.4)	1 (0.4)
Mucosal hemorrhage	1 (0.4)	0.0	0.0
Contusion	1 (0.4)	0.0	0.0
**Interstitial lung disease**			
Patients with ≥1 event	3 (1.2)	1 (0.4)	1 (0.4)
Interstitial lung disease	1 (0.4)	0.0	0.0
Lung disorder	1 (0.4)	1 (0.4)	1 (0.4)
Pulmonary toxicity	1 (0.4)	0.0	0.0

Abbreviations: AE, adverse event; ALP, alkaline phosphatase; ALT, alanine aminotransferase; AST, aspartate aminotransferase; GGT, Gamma-glutamyltransferase; MedDRA, Medical Dictionary for Regulatory Activities; PT, preferred term; SAE, serious adverse event.

^a^The data for liver injury- and hypersensitivity-related AEs include those that occurred in at least 1% of patients.

^b^The terms used to describe the AEs are adapted from or composites of MedDRA Version 27.0, preferred terms.

^c^These are hypersensitivity-related events from CRF and marked as hypersensitivity-related by reporting physician.

##### Hypersensitivity

The specific AE of “hypersensitivity (MedDRA PT)” was reported in 9.9% (n = 24) and no “drug hypersensitivity (MedDRA PT)” was reported. Grade 3 hypersensitivity events occurred in 2.5% of patients (n = 6), and Grade 4 events in 0.8% of patients (n = 2).

**Figure 2:**
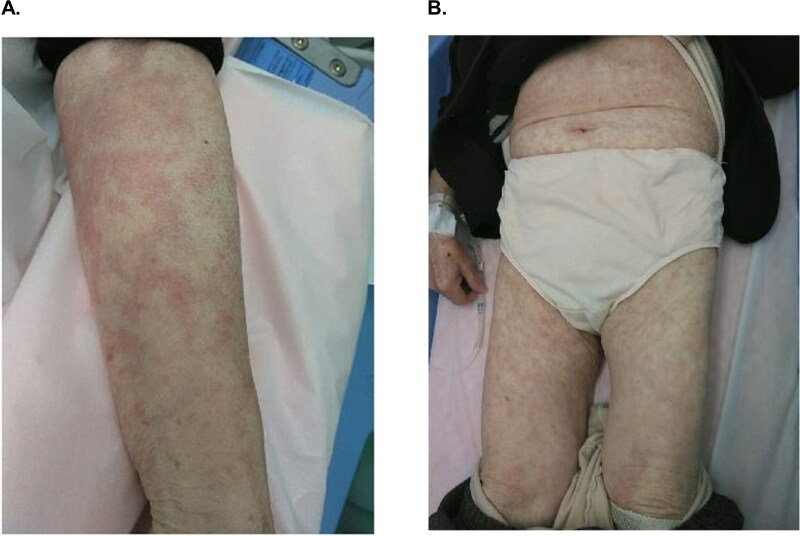
Patient diagnosis for hypersensitivity-related AEs: (A) Macular rash: fused faint erythema, scattered ranging from a few millimeters to about one centimeter on the forearm; (B) Diffuse erythema: ranging from a few millimeters to several centimeters, many are fused. AE, adverse event. Note: To safeguard patient privacy and comply with local regulatory requirements and global ethical guidelines, explicit consent was obtained from patients who experienced selpercatinib hypersensitivity, for the collection and use of their photographs in scientific publications. All photographs have been fully anonymized; each image was carefully reviewed to ensure no identifying features (e.g. distinctive markings or personal items) are visible. Only the skin region affected by the hypersensitivity rash is shown, with all surrounding context removed.

Additionally, hypersensitivity-related events were reported in 14.8% of patients (n = 36), based on eCRF documentation by the treating physician. Among these, rash (5.8% [n = 14]) and abnormal hepatic function (5.3% [n = 13]) were most prevalent **(**Table 4**)**. Overall, 5.8% (n = 14) of patients experienced Grade ≥ 3 hypersensitivity-related events. The median (min-max) time to onset of hypersensitivity-related event was 17.5 days (3–219). Figure 2 illustrates a case of hypersensitivity-related rash in a patient with *RET* fusion-positive NSCLC, bone and lymph node metastases, and multiple comorbidities (hypertension, hemorrhage, and pulmonary artery thrombosis) and treated with selpercatinib. Following treatment interruption, the patient was treated with prednisolone and recovered from the hypersensitivity reaction. Selpercatinib treatment was resumed after recovery.

**Figure 3:**
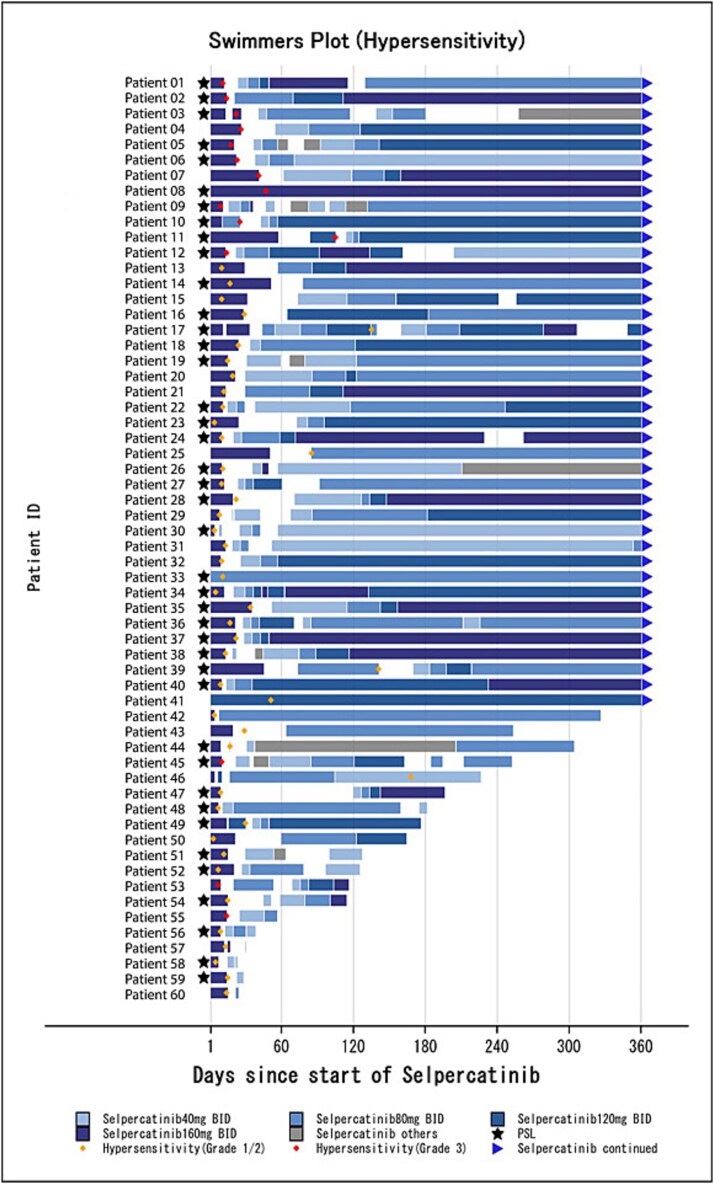
Swimmer plots: Hypersensitivity including hypersensitivity-related events (safety analysis set: cases with hypersensitivity) BID, twice daily.

Of those with hypersensitivity (MedDRA PT) and hypersensitivity-related events, 86.7% (n = 52) of patients had treatment interrupted in alignment with guidance in Japanese package insert [12], and selpercatinib was resumed at reduced doses (40 mg: 70.0% [n = 42], 80 mg: 11.7% [n = 7], 120 mg: 3.3% [n = 2]). One (1.7%) patient had a dose reduction without dose interruption, and 7 (11.7%) patients continued treatment without a dose interruption despite the occurrence of hypersensitivity-related events **(**Supplementary Table S2**)**. Swimmer plots **(**Fig. 3**)** demonstrated that for patients with hypersensitivity-related events, treatment interruptions, prednisolone (or its equivalent) administration, or selpercatinib dose reductions were implemented as needed. Most hypersensitivity and hypersensitivity-related events (95.0% [n = 57/60]) had recovered or were recovering during the observation period.

##### Other adverse event of special interest

A total of 23.9% (n = 58/243) of patients experienced at least one hypertension-related AE of any grade, with 9.9% (n = 24) reporting Grade ≥ 3 events. Most cases (79.3% [n = 46]) recovered during the observation period **(**Table 4**)**. QT prolongation of any grade occurred in 16 of 243 (6.6%) patients, with 4 (1.6%) experiencing Grade ≥ 3, and 1 (0.4%) SAE. No arrhythmias or secondary cardiac events were reported. Overall, 5 (2.1%) patients had hemorrhage-related AEs, 2 of which were SAEs and Grade ≥ 3, including 1 death due to hemoptysis. ILD-related events were reported in 3 patients, including 1 Grade 4 SAE (lung disorder) **(**Table 4**)**.

#### Adverse events by prior immune checkpoint inhibitor use

Of the total 243 patients, 93 (38.3%) had prior ICI treatment. These patients generally experienced fewer AEs, particularly “liver injury” and “hypersensitivity”, compared to those without prior ICI exposure **(**Supplementary Table S3**)**. The incidence of liver injury-related AEs was 52.0% (n = 78/150) in patients without prior ICI treatment versus 31.2% (n = 29/93) in those with prior ICI treatment. Similarly, hypersensitivity (MedDRA PT) and hypersensitivity-related events occurred in 30.7% (n = 46/150) of patients without prior ICI treatment and 15.1% (n = 14/93) in those with prior ICI treatment **(**Supplementary Table S3**)**. Moreover, patients with prior ICI treatment had a lower incidence of SAEs **(**Supplementary Table S4**)** and Grade ≥ 3 AEs (excluding QT interval prolongation and Cardiac arrhythmia due to QT prolongation).

### Effectiveness

#### Tumor response

**Figure 4:**
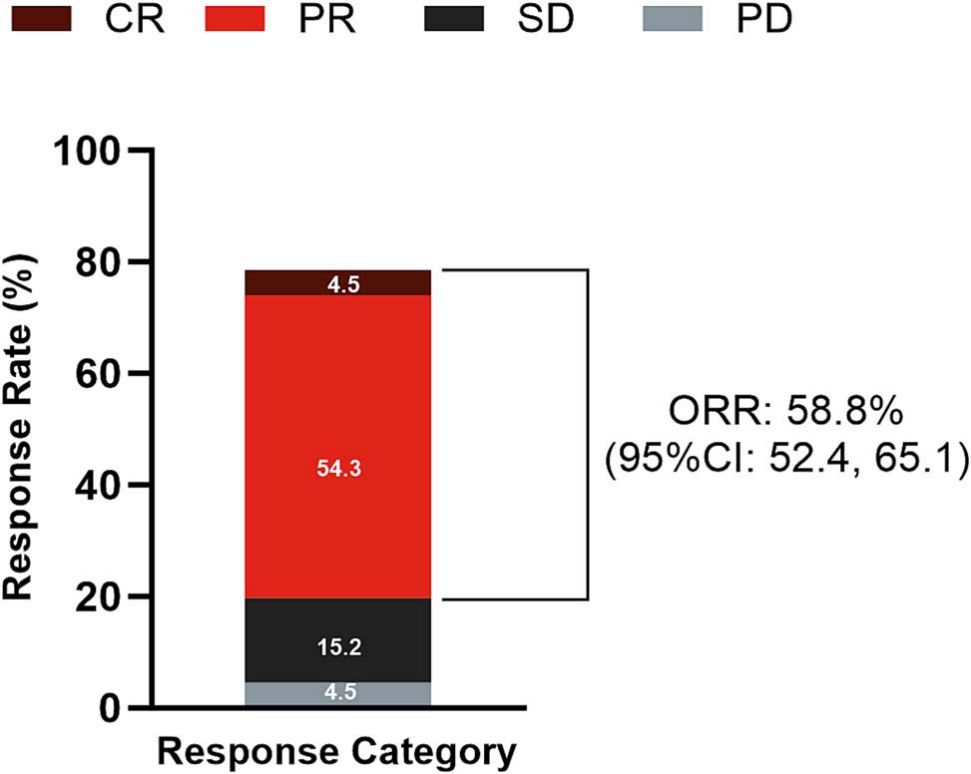
Tumor response for target lesions^a,b^ CI, confidence interval; CR, complete remission, complete response; PD, progressive disease; PR, partial response; SD, stable disease. ^a^Percentages are based on the effectiveness analysis set (N = 243). ^b^Only patients with target lesions (n = 212 [87.2%]) are shown: CR, 11; PR, 132; SD, 37; PD, 11. Patients with not evaluable responses (n = 3) and those with undetermined effectiveness (n = 18) were excluded. Overall response: Best response in 6-month and 12-month. Response rate: ([CR + PR]/the number of effectiveness analysis set) ^*^ 100.

In all, 212 of 243 (87.2%) patients were analyzed for target lesions, and the ORR was 58.8% (95% CI: 52.4–65.1). Of the total 243 patients, 11 (4.5%) achieved a CR, and 132 (54.3%) patients achieved PR **(**Fig. 4**)**. ORR was higher in treatment-naïve patients (69.9% [95% CI: 59.5–79.0]) compared to those with prior systemic anti-cancer therapy (52.0% [95% CI: 43.7–60.2])***.***

#### Survival status

**Figure 5:**
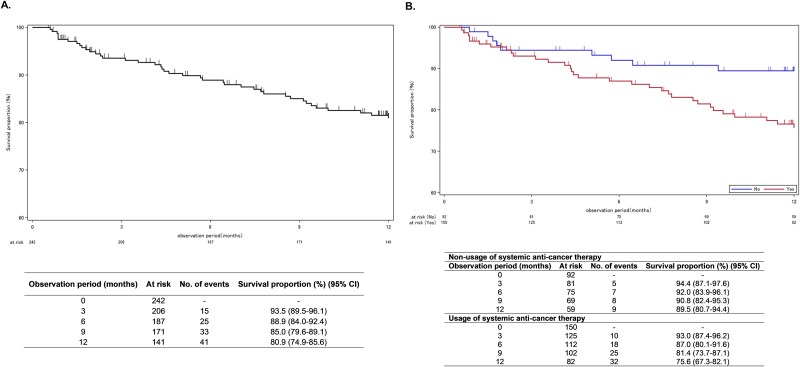
Survival proportions over a 12-month observation period: (A) Effectiveness analysis set; (B) By history of systemic anti-cancer therapy in effectiveness analysis set CI, confidence interval; effectiveness analysis set, N = 243.

During the 12-month observation period, 41 patients died, 32 with a history of systemic anti-cancer therapy and 9 without. For the overall population, the median OS was not reached, with survival rates at 6 and 12 months being 88.9% (95% CI: 84.0–92.4) and 80.9% (95% CI: 74.9–85.6), respectively **(**Fig. 5**)**. In patients with prior therapy, the 6-month and 12-month survival rates were 87.0% (95% CI: 80.1–91.6) and 75.6% (95% CI: 67.3–82.1), respectively. In treatment-naïve patients, the survival rates were 92.0% (95% CI: 83.9–96.1) at 6 months and 89.5% (95% CI: 80.7–94.4) at 12 months.

## Discussion

The findings from this PMSS provide crucial insights into the safety and effectiveness of selpercatinib for patients with *RET* fusion-positive NSCLC in real-world clinical settings in Japan. This study is particularly significant given the limited data available from Japanese patients and the rarity of *RET* fusion-driven cancers.

The safety profile of selpercatinib observed in this study was consistent with that reported in clinical studies [14–16], reinforcing selpercatinib’s therapeutic potential while highlighting key safety considerations. In the present study, the real-world use of selpercatinib was generally consistent with the recommended dosing regimen (160 mg twice daily), with a median RDI of 73.8%.

AEs were reported in 86.0% of patients, with 48.1% experiencing Grade ≥ 3 AEs, and 24.7% reporting SAEs. These rates are lower than those observed in previous clinical studies, including the global phase 3 study (AEs: 100%; Grade ≥ 3 AEs: 70.3%; SAEs: 34.8%) [15] and the subgroup analysis of the phase 1/2 study for Japanese patients (AEs: 100%; Grade ≥ 3 AEs: 50.0%) [16]. This numerical difference is a well-recognized feature of real-world observational studies, arising from differences in monitoring intensity and reporting rigor compared to highly controlled clinical trials. No new safety concerns were identified in the current study. The most frequently reported AEs were hypertension, abnormal hepatic function, rash, diarrhea, AST and ALT elevations, and hypersensitivity, which are consistent with the known safety profile of selpercatinib [13]. SAEs primarily included abnormal hepatic function and hypersensitivity, while common Grade ≥ 3 AEs included abnormal hepatic function, hypertension, ALT increases, and hypersensitivity. These findings were consistent with previous clinical studies, where hypertension, increase in ALT and AST, diarrhea, hypersensitivity, and ECG QT prolongation were common [14, 15]. Given that most AEs were nonserious and the median RDI was 73.8%, selpercatinib appears to have a favorable safety profile in real-world clinical settings.

Hypertension was the most commonly observed AE in this study, affecting 23.5% of patients. Although PMSS are not directly comparable to clinical trials, the incidence of hypertension was lower compared to previous studies [14–16]. Importantly, ~80% of hypertension cases in this study were manageable with dose adjustments or antihypertensive therapy, emphasizing the importance of proactive monitoring and early intervention in managing hypertension in clinical practice.

Similarly, liver injury-related AEs (abnormal hepatic function, AST and ALT increase, etc.) were prominent, affecting 44.0% of patients, with 10.3% experiencing SAEs. These findings concur with prior data, reinforcing liver toxicity as a key safety concern for *RET* inhibitors [21, 24]. However, it is noteworthy that nearly 90% of liver injury-related AEs reported in this study resolved, emphasizing the importance of regular liver function monitoring and potential dose adjustments to mitigate the risk of severe liver injury.

Selpercatinib is associated with delayed hypersensitivity reactions, distinct from immediate hypersensitivity reactions like anaphylaxis [20, 21]. In the current study, both formally reported “hypersensitivity AEs (hypersensitivity and drug hypersensitivity [MedDRA PT]” and physician-identified hypersensitivity-related events were captured. This comprehensive approach enabled a more accurate assessment of selpercatinib’s safety profile.

In this study, the incidence of hypersensitivity AEs was similar to previous clinical studies [14–16], and no new signs or symptoms of hypersensitivity were identified in physician-flagged events not formally reported as AEs. Considering the broader reporting criteria used in this study, combining hypersensitivity AEs and physician-identified hypersensitivity-related events, only one patient discontinued treatment despite the higher frequency. Most events resolved during follow-up, suggesting that selpercatinib-associated hypersensitivity events are generally manageable with appropriate monitoring and do not typically require treatment discontinuation. In this study, most of the hypersensitivity and hypersensitivity-related events (~87%) were managed with dose reductions. In Japan, standard guidance recommends withholding selpercatinib until hypersensitivity symptoms resolve. During this period, systemic corticosteroids (1 mg/kg/day) with appropriate prophylaxis should be initiated and continued until symptom resolution [12]. Selpercatinib may then be resumed at a reduced dose, 3 levels below the dose at which hypersensitivity occurred (dose levels: 120 mg twice-daily [level 1], 80 mg twice-daily [level 2], and 40 mg twice-daily [level 3]), with weekly dose escalation by one level as tolerated, until the target dose is re-established. However, this study observed variability in real-world management, with some patients treated without steroids, and others receiving varying corticosteroid regimens. These findings underscore the importance of individualized management strategies in effectively managing selpercatinib-associated hypersensitivity.

Interestingly, patients with prior ICI treatment had lower incidences of liver injury-related and hypersensitivity-related events, as well as overall SAEs and Grade ≥ 3 AEs, compared to those without prior ICI exposure. This contrasts with earlier clinical study, which reported higher toxicity rates in ICI-pretreated patients [20, 25]. This observation warrants further investigation and may inform sequencing strategies and safety monitoring in clinical practice.

Direct comparisons between observational studies and controlled trials are inherently limited by differences in monitoring intensity and AE reporting sensitivity. However, prior studies have shown that East Asian patients, including Japanese populations, tend to experience higher incidence of specific AEs, such as elevated AST/ALT, hypertension, and QTc prolongation [6, 14–16, 26], as well as more frequent Grade ≥ 3 AEs and SAEs [15]. When AEs such as “liver injury”, “QT interval prolongation”, or “hypersensitivity” occur, timely interventions, including dose reduction, treatment interruption, or discontinuation are essential to ensure patient safety.

This study demonstrates the effectiveness of selpercatinib in a real-world setting. The ORR observed in this study was lower than that reported in the phase 1/2 and phase 3 studies, [14, 15] potentially reflecting differences in evaluation methods, including the absence of standardized imaging intervals used in clinical trials. Additionally, the inclusion of a broader real-world population, such as older patients and those with ECOG PS ≥2, may have contributed to the lower ORR. Selpercatinib demonstrated effectiveness in both treatment-naïve and previously treated patients with *RET* fusion-positive NSCLC, including those who had received second-line or later chemotherapy. These findings reinforce selpercatinib's value as a targeted therapy for *RET* fusion-positive NSCLC, especially when used early in the treatment course, in real-world clinical practice, including in the Japanese population.

### Strengths and limitations

This study included a diverse cohort with varying clinical characteristics and treatment histories, enhancing its relevance to real-world clinical practice in Japan. As treatment decisions were made at physicians’ discretion, the findings reflect routine care, though this limits direct comparability with clinical trials. The noncomparative, observational design limited analyses to descriptive statistics, and subgroup comparisons should be interpreted with caution due to small sample sizes and potential random variation. Japanese regulatory requirements mandated inclusion of all patients treated with selpercatinib during the study period, potentially allowing rare duplicate enrollments when patients changed hospitals. Most baseline data were extracted from medical records, minimizing biases related to recall; however, missing data and underreporting of mild AEs are inherent limitations of observational studies. Additionally, the lower incidence rates of AEs observed in this PMSS, compared to controlled clinical trials, may be attributable to differences in study design, monitoring intensity, and the reduced reporting diligence i.e. typical of real-world observational settings. Finally, the follow-up period was limited to 12 months, which may not have fully captured long-term survival outcomes. However, it was sufficient for comparison with previous evidence.

### Conclusion

This study found no new safety concerns with selpercatinib in patients with *RET* fusion-positive NSCLC in routine clinical practice in Japan. Overall, the treatment showed a manageable safety profile, as evidenced by the low number of discontinuations due to AEs. The most AEs were nonserious and could be managed through dose adjustments, dose withholding, or concomitant medications, reinforcing the established safety profile of selpercatinib. Furthermore, selpercatinib demonstrated tumor response, including in patients with prior chemotherapy, underscoring it as an effective treatment option for *RET* fusion-positive NSCLC across different lines of therapy.

## Supplementary Material

Supplementary_Material_18_Dec_2025_hyaf220

## Data Availability

The datasets generated and analyzed during this study are not publicly available due to the observational nature of the research and the absence of participant or caregiver consent for data sharing.
