# Supplementary material for: Safety and effectiveness of selpercatinib in patients with RET fusion-positive non-small cell lung cancer in real-world clinical practice: a postmarketing study in Japan
Source: Jpn J Clin Oncol. 2026 Jan 25;56(4):435–46. doi: 10.1093/jjco/hyaf220 (PMC13070520; doi:10.1093/jjco/hyaf220)
Supplement: Supplementary_Material_18_Dec_2025_hyaf220 [file supplementary_material_18_dec_2025_hyaf220.docx]

**Supplementary Material**

**Safety and effectiveness of selpercatinib in patients with *RET* fusion-positive non-small cell lung cancer in real‑world clinical practice: A post‑marketing study in Japan**

Yucherng Chen, Joji Mori, Satoshi Wakabayashi, Kevin Y. Urayama*

Eli Lilly Japan K.K., Hyogo, Japan

***Corresponding author:**

Kevin Y. Urayama, PhD, MPH

Eli Lilly Japan K.K., Kobe, Hyogo, Japan

Email: [kevin.urayama@lilly.com](mailto:kevin.urayama@lilly.com)

Phone: +81-80-5383-6349

**Table S1.** Exposure to selpercatinib

| **Variables** | **Total  (N=243)** |
| --- | --- |
| **Actual administration period (days)B** | **n=243** |
| Mean (SD) | 257.1 (126.3) |
| Median (min-max) | 330.0 (1-360) |
| **Actual administration period, n (%)** | **n=243** |
| ≥0 month and <1 month | 26 (10.7) |
| ≥1 month and <3 months | 19 (7.8) |
| ≥3 month and <6 months | 23 (9.5) |
| ≥6 months and **≤**12 months | 175 (72.0) |
| **Total administration period (days)**b | **n=243** |
| Mean (SD) | 275.9 (128.0) |
| Median (min-max) | 360.0 (1-360) |
| **Total administration period, n (%)** | **n=243** |
| ≥0 month and <1 month | 24 (9.9) |
| ≥1 month and <3 months | 14 (5.8) |
| ≥3 month and <6 months | 22 (9.1) |
| ≥6 months and **≤**12 months | 183 (75.3) |
| **Total observational period (days)**c | **n=243** |
| Mean (SD) | 286.5 (115.7) |
| Median (min-max) | 360.0 (31-360) |
| **Total observational period, n (%)** | **n=243** |
| ≥0 month and <1 month | 0 |
| ≥1 month and <3 months | 34 (14.0) |
| ≥3 month and <6 months | 21 (8.6) |
| ≥6 months and **≤**12 months | 188 (77.4) |
| **Relative dose intensity (%)**d | **n=243** |
| Mean (SD) | 70.04 (25.52) |
| Median (min-max) | 73.75 (10.0-100.0) |
| **Drug interruption period (days)**^e^ | **n=141** |
| Mean (SD) | 40.5 (56.4) |
| Median (min-max) | 23.0 (3-353) |
| **Patients with dose reduction(s), n (%)** | **n=243** |
| No dose reduction  1 dose reduction | 108 (44.4)  76 (31.3) |
| 2 dose reductions | 40 (16.5) |
| ≥3 dose reductions | 19 (7.8) |
| **Patients experiencing at least one dose increase after dose reduction, n (%)^f^** | **n=135** |
| Yes | 81 (60.0) |
| No | 54 (40.0) |

aSum of (Administration end date–Administration start date + 1). Set 360 if longer than 360.

bLast administration date–First administration date + 1. Set 360 if longer than 360.

cThe smaller value of [ (Last administration date + 30)–First administration date + 1] or [Subsequent Anti-cancer treatment date–First administration date + 1] or 360.

dRelative dose intensity is calculated as (actual amount of drug taken divided by amount of drug prescribed) *100%.

eFirst drug interruption date–First administration date + 1.

N, number of patients in full analysis set; n, number of patients in the specified category.

Table S2. Dose modification due to liver injury-related and hypersensitivity-related events

| **Liver injury-related events** | |  |
| --- | --- | --- |
| Safety analysis set | | N=243 |
| Item | | n (%) |
| Number of analysis population | | 60 |
| Patients with dose interruption due to ‘liver injury’ with ≥Grade 3 | | 43 (71.7) |
| Patients with dose reduction due to ‘liver injury’ with ≥Grade 3 (no dose interruption) | | 3 (5.0) |
| Patients with dose increase after dose reduction due to ‘liver injury’ with ≥Grade 3 | | 1 (1.7) |
| Maximum daily dose as increased dose after dose reduction | 240 mg | 1 (1.7) |

**Hypersensitivity (PT) and Hypersensitivity related events**

| Safety analysis set | | | N=243 |
| --- | --- | --- | --- |
| Item | | | n (%) |
| Number of analysis population, n | | | 60 |
| Patients with dose interruption due to ‘hypersensitivity’ related events | | | 52 (86.7) |
| Patients who re-administrated with reduced dose after dose interruption | | | 52 (86.7) |
|  | Re-administered dose (one time dose) | 40 mg | 42 (70.0) |
|  |  | 80 mg | 7 (11.7) |
|  |  | 120 mg | 2 (3.3) |
|  |  | 160 mg | 1 (1.7) |
| Patients with dose reduction due to ‘hypersensitivity’ related events (no dose interruption) ^a^ | | | 1 (1.7) |
| Patients without dose modification due to ‘hypersensitivity’ related events (no dose interruption/dose reduction) | | | 7 (11.7) |

MedDRA Version 27.0

CTCAE, Common Terminology Criteria for Adverse Events; MedDRA, Medical Dictionary for Regulatory Activities; N, number of patients in full analysis set; n, number of cases in the specified category; PT, preferred term.

^a^The dose was reduced from 120 mg to 80 mg, then interrupted and re-administrated at 40 mg.

**Table S3.** Adverse events by prior ICI use

| **Safety Analysis Set, N=243** | **Prior ICI Use** | |
| --- | --- | --- |
|  | **No (n=150)** | **Yes (n=93)** |
| Patients with ≥1 any adverse event, n (%) | 111 (74.0) | 46 (49.5) |
| Interstitial lung disease, n (%) |  |  |
| Patients with ≥1 event | 2 (1.3) | 1 (1.1) |
| Pulmonary toxicity | 0 (0.0) | 1 (1.1) |
| ‘Liver injury’, n (%) |  |  |
| Patients with ≥1 event | 78 (52.0) | 29 (31.2) |
| Hepatic function abnormal | 40 (26.7) | 11 (11.8) |
| AST increased | 17 (11.3) | 8 (8.6) |
| ALT increased | 17 (11.3) | 7 (7.5) |
| Liver disorder | 9 (6.0) | 5 (5.4) |
| Hepatic enzyme increased | 4 (2.7) | 0 (0.0) |
| Blood bilirubin increased | 2 (1.3) | 2 (2.2) |
| GGT increased | 2 (1.3) | 1 (1.1) |
| Blood ALP increased | 2 (1.3) | 1 (1.1) |
| Hyperbilirubinemia | 1 (0.7) | 0 (0.0) |
| International normalized ratio increased | 1 (0.7) | 0 (0.0) |
| Transaminases increased | 1 (0.7) | 1 (1.1) |
| Drug-induced liver injury | 1 (0.7) | 0 (0.0) |
| Liver function test increased | 0 (0.0) | 1 (1.1) |
| Ascites | 0 (0.0) | 1 (1.1) |
| Hypoalbuminemia | 0 (0.0) | 1 (1.1) |
| ‘QT interval prolongation’, n (%) |  |  |
| Patients with ≥1 event | 9 (6.0) | 7 (7.5) |
| Electrocardiogram QT prolonged | 9 (6.0) | 7 (7.5) |
| Cardiac arrhythmia due to QT prolongation, n (%) |  |  |
| Patients with ≥1 event | 9 (6.0) | 7 (7.5) |
| Electrocardiogram QT prolonged | 9 (6.0) | 7 (7.5) |
| ‘Hypersensitivity (PT)’ and ‘Hypersensitivity-related events’, n (%) |  |  |
| Patients with ≥1 event | 46 (30.7) | 14 (15.1) |
| Hypersensitivity | 17 (11.3) | 7 (7.5) |
| Rash | 13 (8.7) | 1 (1.1) |
| Hepatic function abnormal | 8 (5.3) | 5 (5.4) |
| Pyrexia | 8 (5.3) | 2 (2.2) |
| Platelet count decreased | 4 (2.7) | 2 (2.2) |
| ALT increased | 2 (1.3) | 1 (1.1) |
| Rash maculopapular | 2 (1.3) | 1 (1.1) |
| AST increased | 1 (0.7) | 1 (1.1) |
| Liver disorder | 1 (0.7) | 1 (1.1) |
| Blood creatinine increased | 0 (0.0) | 1 (1.1) |
| Stomatitis | 0 (0.0) | 1 (1.1) |
| ‘Hypertension’, n (%) |  |  |
| Patients with ≥1 event | 40 (26.7) | 18 (19.4) |
| Hypertension | 39 (26.0) | 18 (19.4) |
| ‘Hemorrhage’, n (%) |  |  |
| Patients with ≥1 event | 3 (2.0) | 2 (2.2) |
| Hemoptysis | 0 (0.0) | 1 (1.1) |
| Contusion | 0 (0.0) | 1 (1.1) |

MedDRA Version 27.0

AE, adverse event; ALP, alkaline phosphatase; ALT, alanine aminotransferase; AST, aspartate aminotransferase; GGT, gamma-glutamyl transferase; ICI, Immune Checkpoint Inhibitor; MedDRA, Medical Dictionary for Regulatory Activities’ PT, preferred term.

**Table S4.** Serious adverse events by prior ICI use

| **Safety Analysis Set, N=243** | **Patients with Prior ICI Use** | |
| --- | --- | --- |
|  | No | Yes |
| Number for safety analysis set | 150 | 93 |
| Patients with >=1 event | 28 | 13 |
| Interstitial lung disease |  |  |
| Patients with >=1 event | 1 (0.7) | 0 |
| Liver injury |  |  |
| Patients with >=1 event | 18 (12.0) | 7 (7.5) |
| Hepatic function abnormal | 14 (9.3) | 4 (4.3) |
| ALT increased | 2 (1.3) | 2 (2.2) |
| AST increased | 2 (1.3) | 1 (1.1) |
| Liver function test increased | 0 | 1 (1.1) |
| Hypersensitivity (PT) and Hypersensitivity-related events |  |  |
| Patients with >=1 event | 11 (7.3) | 6 (6.5) |
| Hypersensitivity | 5 (3.3) | 4 (4.3) |
| Rash | 1 (0.7) | 1 (1.1) |
| Hepatic function abnormal | 4 (2.7) | 1 (1.1) |
| Hypertension |  |  |
| Patients with >=1 event | 1 (0.7) | 1 (1.1) |
| Hypertension | 1 (0.7) | 1 (1.1) |
| Hemorrhage |  |  |
| Patients with >=1 event | 1 (0.7) | 1 (1.1) |
| Hemoptysis | 0 | 1 (1.1) |

MedDRA Version 27.0

ALT, alanine aminotransferase; AST, aspartate aminotransferase; ICI, Immune Checkpoint Inhibitor; MedDRA, Medical Dictionary for Regulatory Activities; SAE, serious adverse event.
